# Willingness‐to‐Pay for Health Insurance in South Asia: A Systematic Review and Meta‐Analysis

**DOI:** 10.1002/puh2.70394

**Published:** 2026-09-29

**Authors:** Md Fuad Al Fidah, Sayeda Sumaiya Efa, Md Ridwan Islam, Md Abdullah Saeed Khan

**Affiliations:** ^1^ National Institute of Preventive and Social Medicine Dhaka Bangladesh; ^2^ BADAS TB Initiative Diabetic Association of Bangladesh (BADAS) Dhaka Bangladesh; ^3^ International Center for Diarrhoeal Disease Research, Bangladesh Dhaka Bangladesh

**Keywords:** health insurance, meta‐analysis, South Asia, willingness‐to‐pay (WTP)

## Abstract

**Introduction:**

In low‐ and middle‐income countries, healthcare systems rely heavily on out‐of‐pocket payments, causing financial hardship and limiting access to care. Globally, 800 million people spend over 10% of household income on healthcare, driving 100 million into extreme poverty due to catastrophic health expenditures (CHE). In South Asia, out‐of‐pocket (OOP) payments remain the primary funding source, with India and Bangladesh reporting significant CHE burdens. Health insurance (HI) can reduce costs, improve access and mitigate CHE, playing a key role in achieving universal health coverage.

**Objective:**

We conducted this systematic review and meta‐analysis to estimate the proportion of willingness‐to‐pay (WTP) for HI and to explore the factors generally associated with it.

**Methods:**

Electronic searches of PubMed, Google Scholar and Bangladesh Journals Online (BanglaJOL), as well as citation tracking, were conducted to identify eligible studies. Study quality was assessed using the Newcastle‐Ottawa Scale. Publication bias was evaluated using Egger's test and funnel plots. Leave‐one‐out sensitivity and subgroup analyses by WTP elicitation method and insurance type were conducted. All analyses were conducted in STATA 19.

**Results:**

We included 17 studies from 5 South Asian countries in the analysis. The pooled proportion of WTP for HI was 0.72 (95% CI: 0.61−0.82), with a 95% prediction interval of 0.20−1.00 and considerable heterogeneity (*I* ^2^ = 99.59%, *τ* ^2^ = 0.2474). The pooled proportion from Bangladeshi studies was higher than that of Indian studies (0.83 and 0.69, respectively). Leave‐one‐out estimates ranged from 0.70 to 0.76. Age, sex, level of education, size of the family, socio‐economic status, monthly personal income, occupation, family income, healthcare utilisation and healthcare expenditure were generally associated with WTP.

**Conclusion:**

Overall, 72% of participants were willing to pay for HI, although substantial heterogeneity was observed across studies. Further robust studies are needed to better characterise WTP for HI in South Asia.

## Graphical abstract

Seventeen studies from five South Asian countries were included. The pooled proportion willing to pay for health insurance was 72% (95% CI: 61%–82%), with higher estimates in Bangladesh than India. Age, education, income, occupation, socio‐economic status, healthcare utilisation and healthcare expenditure were commonly associated with willingness‐to‐pay.

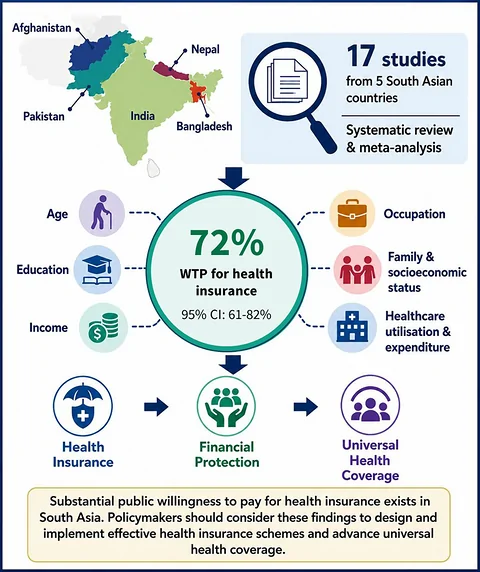

## 1 Introduction

In low‐ and middle‐income countries (LMICs), healthcare systems heavily rely on out‐of‐pocket (OOP) payments as their main funding source. This places a substantial financial strain on individuals, especially those who are economically vulnerable. The cost of medical care not only depletes personal finances but also creates barriers to fair access to healthcare services [1]. Globally, around 800 million people allocate more than 10% of their household income to medical expenses, and as a result, catastrophic health expenditures (CHE) have driven 100 million people into extreme poverty [2].

Recent studies have increasingly examined the effects of OOP payments on CHE and poverty across various Asian countries [3, 4, 5]. One study conducted in 2018 found that, in Southeast Asia, 242.7 million people faced CHE at a threshold of 10%, and 58.2 million were pushed below the poverty line [6]. South Asia, which is one of the world's most densely populated regions with diverse geography, comprises Afghanistan, Bangladesh, Bhutan, India, the Maldives, Nepal, Pakistan and Sri Lanka [7]. Many South Asian countries face a double burden of disease and limited health service coverage, with OOP payments being the primary funding source. Due to inadequate public funding and limited access to health insurance (HI), disadvantaged populations struggle to afford care. This lack of insurance and reliance on personal payments can lead to financial hardship, asset depletion and reduced access to essential health services [8]. In India, OOP payments account for 48.8% of healthcare costs, highlighting the significant financial burden on households as reported by a study in 2024 [9]. In Bangladesh, another LMIC in South Asia, 17% of the population is affected by CHE [10]. HI can be an important tool that can protect individuals from CHE. The primary aim of the HI system is to enhance access to healthcare services, reduce healthcare costs, and mitigate the occurrence of CHE. Additionally, universal health coverage (UHC), which is one of the primary objectives for health policymakers globally, considers HI as the key strategy [11].

HI plays a crucial role in transferring funds from healthy individuals to cover medical costs, making healthcare more affordable and accessible while reducing the financial burden of illness [12]. It pools contributions or taxes to finance medical expenses and includes various models, such as public, community‐based and private insurance [13]. A key concept in HI is the willingness‐to‐pay (WTP), which reflects whether an individual is interested in paying a premium for HI. Moreover, the maximum amount an individual is willing to spend on HI is often influenced by income, healthcare needs, risk preferences and so on [14]. WTP reflects respondents’ stated WTP for enrolment in a HI scheme and, in some studies [15], the maximum amount they were willing to pay. It is based on welfare economics and utility theory, where individuals aim to maximise their well‐being within a limited income [14]. In health economics, WTP helps policymakers design sustainable insurance schemes by showing what people can afford and what kind of benefits they prefer. It avoids underpricing or overpricing that can reduce enrolment [16]. Among the various WTP measurement methods, contingent valuation method (CVM) is widely used in LMICs due to the absence of formal markets. The double‐bound dichotomous choice (DBDC) approach in CVM provides more accurate WTP estimation [17, 18].

To successfully design HI programmes for achieving UHC, it is essential to understand the proportion of people who are willing‐to‐pay for HI [19]. The aim of this systematic review and meta‐analysis was to estimate the proportion of WTP for HI in South Asian countries and to explore the factors generally associated with it.

## 2 Methods

### 2.1 Information Sources

We conducted a literature search during January and February 2024 and also included grey literature. It was conducted following the recommendation of the PRISMA (Preferred Reporting Items for Systematic Reviews and Meta‐Analysis) guidelines. Relevant articles were searched in the PubMed, Google Scholar and the Bangladesh Journals Online (BanglaJOL) websites. As we focused on South Asia, we included BanglaJOL, a local journal platform, to capture regionally published studies. We also searched Google Scholar, which includes many peer‐reviewed and grey literature sources that are often missed by PubMed. Additionally, we used citation tracking to make our search more comprehensive and avoid missing important studies. Other databases like Web of Science, EMBASE and CINAHL were not searched as they are paid‐access databases. This review was not prospectively registered. However, a pre‐defined eligibility criterion and a structured process for study selection, data extraction and quality assessment were established before conducting the review. The search was conducted intensively over a 2‐month period (January and February 2024). The scope of the review was defined narrowly to studies from South Asia with reported WTP proportions, allowing a focused search process.

### 2.2 Search Strategy

The search strategy employed by the study was developed by one author and reviewed by the other authors. Two authors conducted the search individually. They screened all titles, abstracts and full texts. If consensus was not achieved, the senior author made the final decision. Our search was limited to the English language for practical reasons and focused on eight countries of South Asia: Afghanistan, Bangladesh, Bhutan, India, Maldives, Nepal, Pakistan and Sri Lanka. The complete search strategy is provided in Table S1.

### 2.3 Study Selection

We included original, peer‐reviewed quantitative studies, as well as relevant grey literature (e.g., local journal articles and one unpublished thesis), provided they met the eligibility criteria and passed quality assessment. These sources were primarily cross‐sectional in design and were conducted in South Asian countries and reported the proportion of individuals or households WTP for any type of HI. Only articles published in English language were considered.

We excluded commentaries, editorials, reviews, book chapters and conference abstracts. Studies using qualitative methods only or those that did not provide extractable WTP data were also excluded. Additionally, studies conducted outside South Asia or those that received an unsatisfactory quality score (≤4 stars) on the Newcastle‐Ottawa Scale (NOS) were excluded from the final review. We did not set a time limit for publication because WTP studies in South Asia are limited, and we wanted to capture all available data.

### 2.4 Data Extraction

After the exclusion of duplicate studies and including grey literature identified using citation tracking, two authors independently evaluated the potentially eligible studies. In the first step, articles were screened by reviewing the title and the abstract. If multiple HI schemes were reported, the highest proportion was considered to provide a consistent estimate across studies and to avoid selecting an insurance scheme based on subjective judgement. For studies using more than one elicitation method, the study was classified according to the primary method used to elicit WTP, with the method applied first in the elicitation sequence determining the subgroup classification. Any disagreement was resolved through consensus. After screening, two authors independently read the eligible articles. The extracted data included first author, publication year, country, sample size, number of participants with WTP, beneficiary (individual/household), residence (urban/rural) and variables affecting the WTP. The study by Al Fidah et al. was identified from a 2024 thesis, which was subsequently published as a journal article in 2025.

### 2.5 Quality Assurance

We employed four steps to ensure quality. At first, all duplicates were eliminated. Then the remaining studies were screened using the title and the abstract, and all unrelated studies were removed. In the third step, two authors independently assessed the full texts of the remaining articles and excluded unrelated studies. Finally, we conducted a critical appraisal of the remaining studies using the NOS adopted for cross‐sectional studies (Table S2). The NOS considers three aspects of each study and assigns stars based on criteria. Studies having unsatisfactory grades (0–4 stars) were not included in our systematic review. This assessment was done independently by two reviewers. All differences of opinion were resolved by consensus. If a consensus was not achieved, the senior author made the final decision. For the study authored by members of the review team, screening, data extraction and quality appraisal were independently conducted by review authors who were not involved in the study. Three studies were excluded after quality appraisal because their NOS scores were ≤4 (Table S2) [20, 21, 22].

### 2.6 Statistical Analysis

Meta‐analysis was performed using the official meta‐analysis suite in STATA 19 (StataCorp LLC, College Station, TX, USA) using a random‐effects model with restricted maximum likelihood (REML) estimation and the Freeman–Tukey double arcsine transformation. We conducted a meta‐analysis synthesis to assess the proportion of participants with WTP for HI, and vote counting was used to identify factors affecting the WTP [23]. Meta‐analysis was also conducted for prespecified or data‐driven subgroups according to country, beneficiary unit, residence, insurance type and WTP elicitation method. Differences between subgroup estimates were assessed using the test for subgroup differences provided by the meta‐analysis procedure. For each meta‐analysis, we calculated the *I* ^2^ statistics. Heterogeneity was assessed using Cochran's *Q*‐test, *τ* ^2^, and *I* ^2^ statistics. *I* ^2^ values of approximately 50% were considered indicative of substantial heterogeneity, whereas values of ≥75% were considered indicative of considerable heterogeneity. The proportion of WTP (with 95% confidence interval [CI]) was also reported. We assessed funnel‐plot asymmetry using a funnel plot and Egger's regression test; these assessments were interpreted cautiously because of the known limitations of these methods for proportion data.

For the vote‐counting approach, we identified variables significantly associated with WTP that were reported by the studies [23]. As some studies only used the chi‐square test of association, the nature of the effect (positive/negative) of these variables was not considered for all participating studies. If a study reported such an association, it was considered a ‘vote’ for that respective variable. We considered having a minimum of three votes as the criterion of a variable likely affecting WTP for HI. A leave‐one‐out sensitivity analysis was performed by sequentially omitting each study and recalculating the pooled proportion to evaluate the robustness of the overall estimate.

## 3 Results

### 3.1 Study Selection

From electronic database searching, we identified 53 records. An additional 3 records were identified through citation tracking and the authors’ knowledge, resulting in 56 records. After the removal of 8 duplicates, 48 records were screened by title and abstract, of which 21 were excluded. The remaining 27 reports were sought for retrieval, of which 1 could not be obtained. Thus, 26 reports were assessed for eligibility. Following full‐text assessment, nine reports were excluded, including six because of inappropriate study type and three because of an NOS score ≤4. Seventeen studies were included in the systematic review and meta‐analysis. The entire screening process was performed manually by two reviewers. The study selection process is presented in Figure 1.

**FIGURE 1:**
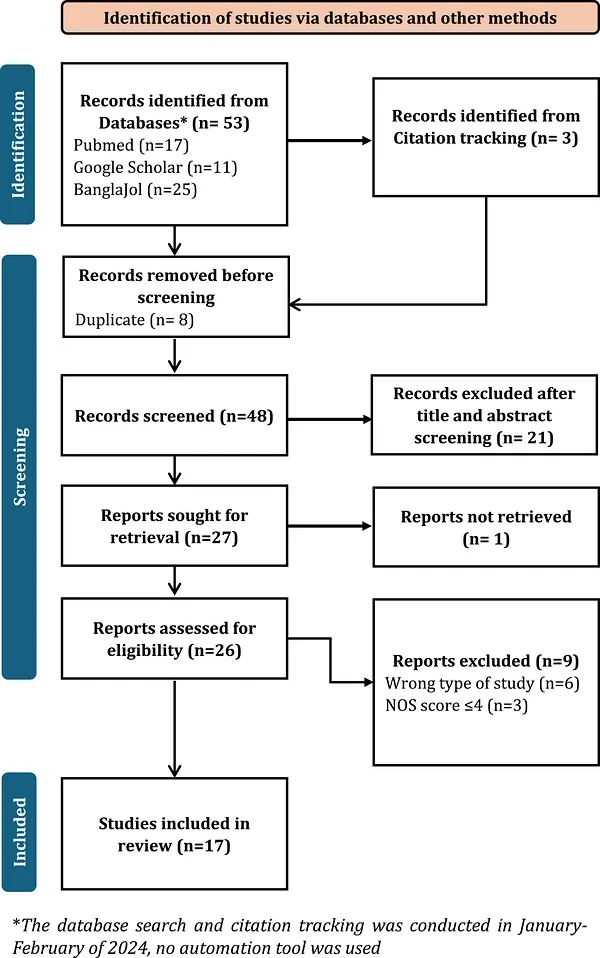
PRISMA 2020 flow diagram of study selection. BanglaJOL, Bangladesh Journals Online; NOS, Newcastle‐Ottawa Scale.

### 3.2 Study Characteristics

The characteristics of the studies included are presented in Table 1.

**TABLE 1:** Characteristics of included studies and willingness‐to‐pay assessment.

Sl. no.	Author (reference)	Country	Beneficiary unit	Residence	Sample size	No of participants reporting WTP	Type of insurance	WTP elicitation method	WTP definition
1.	Ahmed et al. [24]	Bangladesh	Household	Urban	557	483	CBHI	Bidding game	Maximum weekly premium for a CBHI package covering four household members
2.	Al Fidah et al. [18]	Bangladesh	Individual	Urban	232	185	CBHI	DBDC	Willingness‐to‐pay any positive amount for enrolment in CBHI; mean WTP additionally estimated as a percentage of monthly personal income
3.	Alam et al. [17]	Bangladesh	Household	Both	210	195	SHI	DBDC	Maximum WTP for an improved health‐insurance package for the household
4.	Bawa and Ruchita [25]	India	Individual	Both	563	54	HI	Open‐ended question	Willingness to buy health insurance, without elicitation of a maximum monetary amount
5.	Behzad et al. [26]	Afghanistan	Individual	Both	2392	2210	HI	DBDC	Willingness to enrol in the proposed health‐insurance package; maximum annual WTP was subsequently elicited
6.	Dror and Radermacher [27]	Nepal	Individual	Both	2008	920	CBHI	Bidding game	Maximum WTP expressed as amount payable per person per month
7.	Dror et al. [28]	India	Household	Rural	3024	2014	Micro HI	Bidding game	Maximum household WTP for health insurance, expressed as an amount and as a percentage of annual household income
8.	Ghosh [29]	India	Individual	Rural	200	192	HI	Bidding game	Maximum annual premium respondents were willing to pay
9.	Gupta and Trivedi [30]	India	Individual	Both	987	760	HI	DBDC followed by open‐ended	Maximum annual WTP for an individual health‐insurance policy
10.	Kumar et al. [31]	India	Individual	Urban	500	414	SHI	Open‐ended question	Maximum annual premium respondents were willing to pay
11.	Mathiyazhagan [32]	India	Household	Rural	1000	797	CBHI	Bidding game	Maximum amount households were willing to pay for the proposed insurance scheme; 797 positive WTP responses derived from 86.82% of the 918 households willing to join
12.	Mohsin et al. [33]	Bangladesh	Individual	Urban	400	332	HI	Bidding game	Highest monthly premium accepted by the respondent
13.	Rahman et al. [34]	Bangladesh	Individual	Urban	3949	2667	HI	Bidding game	Willingness‐to‐pay a monthly health‐insurance premium; median monthly premium was BDT 15.5
14.	Sana et al. [35]	Pakistan	Household	Urban	200	128	CBHI	Open‐ended question	Willingness‐to‐pay for a community‐based health‐insurance scheme and stated premium amount
15.	Sarwar and Qureshi [36]	Pakistan	Household	Both	380	215	HI	Open‐ended question	Willingness to purchase private health insurance for self/family; respondents also reported the premium amount they were willing to pay
16.	Ullah et al. [37]	Pakistan	Individual	Urban	110	63	Maternal HI	Open‐ended question	Binary interest in accepting maternal health insurance; premium willingness was also assessed
17.	Vanithamani [38]	India	Individual	Urban	1202	757	HI	Open‐ended question	Willingness to participate/pay for a health‐insurance scheme; no maximum monetary WTP amount was elicited

*Note:* Beneficiary unit refers to the unit for which willingness‐to‐pay was elicited or expressed, rather than necessarily the sampling unit.

Abbreviations: CBHI, community‐based health insurance; DBDC, double bound dichotomous choice; HI, health insurance; SHI, social health insurance.

The current review includes studies from five South Asian countries: Bangladesh (*n* = 5) [17, 18, 24, 33, 34], India (*n* = 7) [25, 28, 29, 30, 31, 32, 38], Pakistan (*n* = 3) [35, 36, 37], Afghanistan (*n* = 1) [26] and Nepal (*n* = 1) [27]. The sample sizes of the studies varied greatly, the minimum being 110 and the maximum being 3949. Most (*n* = 11) of the studies offered insurance for individuals, whereas six offered HI for households. Eight studies were conducted on urban, three on rural and six on both urban and rural populations. The most common type of HI offered was not classified, and we considered it ‘Health Insurance’. The second most common type was community‐based HI (CBHI).

### 3.3 WTP for HI

For the meta‐analysis, WTP was operationalised as the proportion of participants reporting positive WTP for the offered HI scheme. Participants reporting WTP were classified as ‘Yes’, whereas those not willing to pay were classified as ‘No’. All 17 studies provided data to conduct a meta‐analysis on the proportion of WTP. A total of five studies were from Bangladesh and seven from India, which was enough for subgroup analysis. Similarly, subgroup analysis was conducted using eight studies on urban residence and five studies where CBHI was offered.

To analyse the overall proportion of WTP for HI and the subgroup estimates, a random‐effects model was used because statistical heterogeneity exceeded the predefined threshold. The pooled proportion of WTP for HI was 0.72 (95% CI: 0.61–0.82), with a 95% prediction interval of 0.20–1.00 and considerable heterogeneity (*I* ^2^ = 99.59%, *τ* ^2^ = 0.25, *p* < 0.001) (Figure 2). In the subgroup analyses, the pooled proportion was 0.69 (95% CI: 0.45–0.88) in India, 0.83 (95% CI: 0.74–0.90) in Bangladesh, 0.74 (95% CI: 0.66–0.81) among urban residents, and 0.72 (95% CI: 0.57–0.85) for CBHI. Considerable heterogeneity was observed within all subgroups, with *I* ^2^ values of 99.75% (*τ* ^2^ = 0.42, *p* < 0.001) in India, 97.14% (*τ* ^2^ = 0.06, *p* < 0.001) in Bangladesh, 97.63% (*τ* ^2^ = 0.06, *p* < 0.001) among urban residents, and 98.85% (*τ* ^2^ = 0.13, *p* < 0.001) for CBHI (Figure S1). No statistically significant funnel‐plot asymmetry was detected (Egger's test, *p* = 0.645) (Figure 2). Leave‐one‐out sensitivity analysis demonstrated that the sequential exclusion of individual studies did not materially alter the pooled proportion estimate (range: 0.70–0.76), indicating that no single study disproportionately influenced the overall pooled estimate (Figure S2). Subgroup analysis by WTP elicitation method demonstrated pooled proportions of 0.77 (95% CI: 0.63–0.88) for the bidding game, 0.86 (95% CI: 0.77–0.93) for DBDC and 0.55 (95% CI: 0.33–0.76) for open‐ended questions. The differences between elicitation methods were statistically significant (test for subgroup differences: *p* = 0.02). Considerable heterogeneity remained within all elicitation‐method subgroups: bidding game (*I* ^2^ = 99.55%, *τ* ^2^ = 0.16, *p* < 0.001), DBDC (*I* ^2^ = 97.44%, *τ* ^2^ = 0.06, *p* < 0.001) and open‐ended questions (*I* ^2^ = 99.26%, *τ* ^2^ = 0.30, *p* < 0.001) (Figure S3).

**FIGURE 2:**
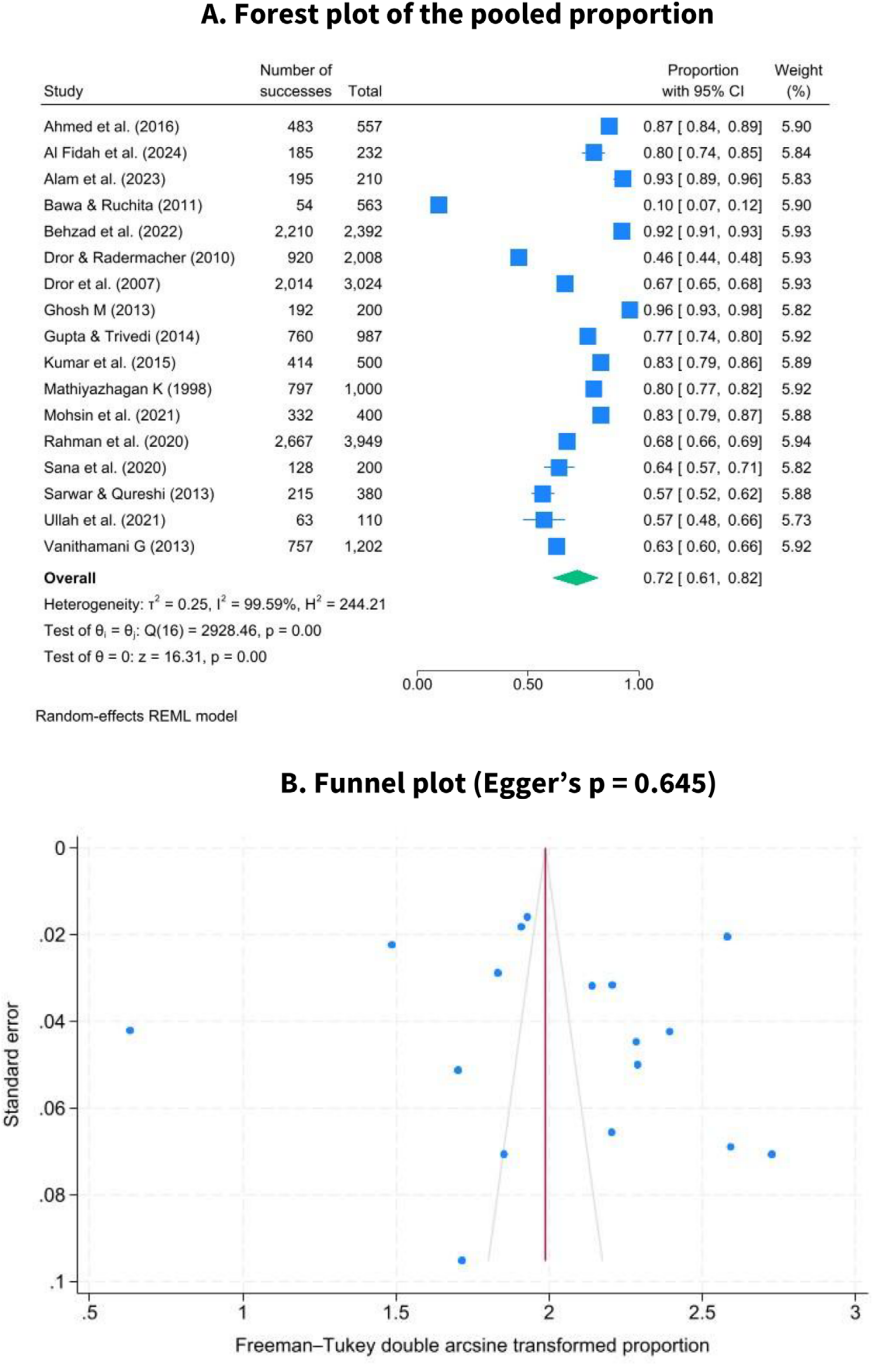
Overall meta‐analysis of willingness‐to‐pay for health insurance: (A) forest plot of the pooled proportion; (B) funnel plot for publication bias, showing the Freeman–Tukey double arcsine transformed proportion against standard error. CBHI, community‐based health insurance; CI, confidence interval; HI, health insurance; WTP, willingness‐to‐pay.

### 3.4 Variables Influencing WTP for HI

The variables that may have a likely effect on WTP were categorised into the following groups:

*Sociodemographic determinants:* Age (*n* = 7) [17, 25, 26, 30, 32, 34, 37], sex (*n* = 4) [25, 26, 28, 30], level of education (*n* = 4) [25, 28, 35, 38], family size (*n* = 3) [17, 32, 34] and socio‐economic status (SES) (*n* = 4) [30, 32, 34, 38] were associated with WTP (Figure 3). Our findings suggest that increasing age was negatively associated with WTP in five studies, whereas male sex was mostly (*n* = 3) positively associated. A positive association with WTP was also found with increasing size of the family (*n* = 2) and higher SES (*n* = 3) by the participating studies.

**FIGURE 3:**
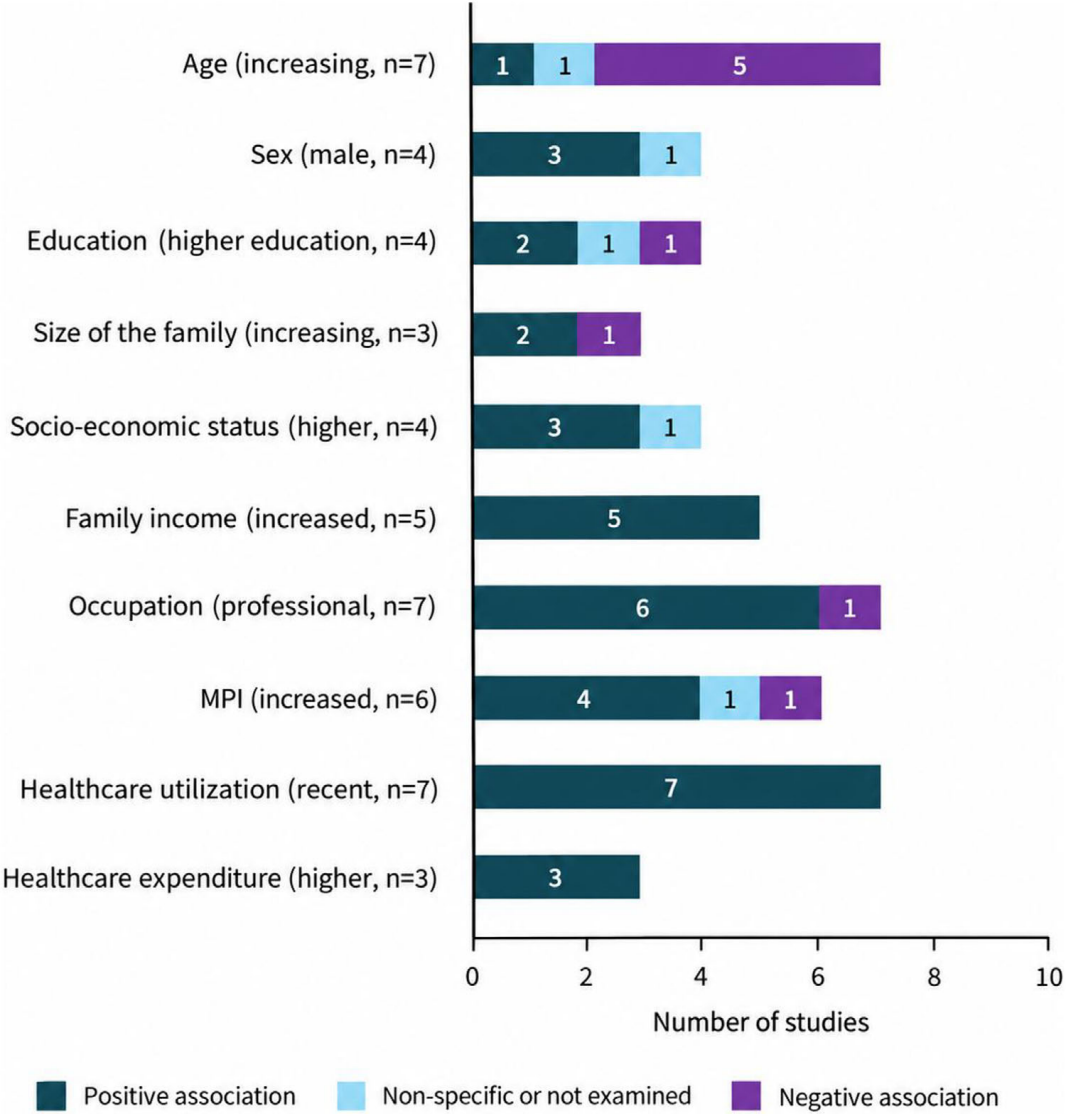
Variables influencing the willingness‐to‐pay for health insurance. MPI, monthly personal income.

*Economic determinants:* We found that family income (*n* = 5) [24, 28, 32, 34, 38], occupation (*n* = 7) [24, 25, 27, 29, 30, 34, 38] and monthly personal income (MPI) (*n* = 6) [25, 28, 29, 35, 37, 38] were most likely to be associated with WTP (Figure 3). A positive association between WTP and family income was reported by all five studies, whereas a similar association was found with MPI by four studies. Reporting professional occupations mostly (*n* = 6) positively influenced the WTP for HI.

*Healthcare‐related determinants:* The healthcare‐related determinants associated with WTP were healthcare utilisation (*n* = 7) [18, 30, 31, 32, 34, 36, 37] and healthcare expenditure (*n* = 3) [28, 31, 36] (Figure 3). Our study found that recent healthcare utilisation (*n* = 7) and higher healthcare expenditure (*n* = 3) were positively associated with WTP for HI.

The methodological quality of the included studies ranged from 5 to 7 on the NOS for cross‐sectional studies, with 14 of the 17 studies scoring ≥6. Detailed quality assessment scores are provided in Table S2.

Subgroup analysis by insurance type showed considerable heterogeneity within the multi‐study categories, with *I* ^2^ values of 92.89% for SHI, 98.85% for CBHI and 99.78% for HI. The pooled WTP proportions were 0.88 (95% CI: 0.77–0.96) for SHI, 0.72 (95% CI: 0.57–0.85) for CBHI and 0.70 (95% CI: 0.48–0.88) for HI. The test for differences between insurance‐type subgroups was statistically significant (*p* = 0.003), although this test included two singleton subgroups, maternal HI and micro HI. Maternal HI and micro HI were each represented by a single study (Figure S4).

## 4 Discussion

This was one of the first systematic reviews and meta‐analysis of WTP of individuals or households for HI in South Asian countries. After searching electronic sources and using citation tracking, we identified 17 distinct studies from 5 out of the 8 South Asian countries. The objectives of this study were to estimate the proportion of people with WTP for HI and identify the variables that were largely associated with it.

### 4.1 Pooled Proportions of WTP for HI

The results of the meta‐analysis suggest that the total proportion of WTP for HI was 0.72, which is higher than that of India but lower than the Bangladeshi estimate. Additionally, urban residents also reported a higher level of proportion. Various studies have reported a range of proportions regarding WTP for HI in South Asia. This ranges from as low as 0.09 in India [25], to 0.93 in Bangladesh [17]. The assessment of publication bias should be interpreted cautiously because, for proportion data, the standard error is mathematically related to the observed proportion itself. Consequently, funnel‐plot asymmetry and Egger's regression may be difficult to interpret reliably, irrespective of the number of studies. The non‐significant Egger's test therefore should not be interpreted as evidence that publication bias or small‐study effects were absent. It may be due to the fact that in Bangladesh, there is no strong and organised electronic source that hosts studies and grey literatures. As such, we might have missed studies that could have affected the results. So, results from this meta‐analysis should be interpreted cautiously. We also found that urban residents reported a higher proportion than the pooled proportion. Urban residents are more likely to have a better understanding of HI, not to mention risk management, which may influence their decision [23]. It may also be possible that urban individuals tend to have higher income and higher SES, which may encourage them towards HI [23]. The pooled WTP proportion for CBHI was similar to the overall estimate. Despite the substantial between‐study heterogeneity, the leave‐one‐out sensitivity analysis demonstrated that the pooled estimate remained stable following sequential exclusion of individual studies, indicating that no single study disproportionately influenced the overall estimate. However, the pooled proportion of 0.72 should be interpreted as the centre of a widely dispersed distribution of study‐specific estimates, rather than as a common underlying proportion, given the high heterogeneity and wide 95% prediction interval (0.20–1.00). WTP estimates differed significantly across elicitation methods; however, considerable residual heterogeneity remained within each subgroup.

The pooled proportion of WTP was 0.83 among studies from Bangladesh and 0.69 among studies from India. This descriptive difference should be interpreted cautiously, as no formal test of between‐country differences was conducted and the CIs overlapped. In Bangladesh, less than 1% of citizens hold some form of HI [39]. The limited availability of HI in the country may be relevant to the observed WTP estimate, although this cannot be established from the present analysis.

### 4.2 A Review of Factors Associated With WTP

Several sociodemographic, economic and healthcare‐related variables were reported to be associated with WTP. Age was significantly associated with WTP for HI, as were sex, level of education, size of the family and SES. Mathiyazhagan [32] reported a negative association of WTP with older individuals, who expressed a lower level of willingness, as did Behzad et al. [26], Ullah et al. [37], Rahman et al. [34] and Gupta and Trivedi [30]. Only Alam et al. reported that a 1‐year increase in age also increases the WTP for HI [17]. Although Bawa and Ruchita reported an association, they did not mention whether this association was positive or negative [25]. In their systematic review, Nosratnejad et al. also noted that older age was associated with lower WTP, whereas larger family size and better education increased WTP, which matches our subgroup analysis [23]. A study conducted in Cameroon reported a higher WTP for HI among younger participants [40]. It has been suggested that, in recent years, the impact of older age on healthcare has been lessened [41]. Additionally, another study argues that older people might have less capacity to pay for HI [42]. These factors can explain the increased WTP among younger people.

Sex was reported to be associated with WTP by four studies. Bawa and Ruchita reported that this association, although present, were not strong [25]. Males being more willing‐to‐pay compared to female were reported by Behzad et al. [26], Dror et al. [28] and Gupta and Trivedi [30]. However, Miti et al. argued that although men tend to be more willing‐to‐pay for HI, the association is weak [43]. As men are often the head of the household and enjoys financial decision‐making power, this finding is understandable.

Bawa and Ruchita reported that they found a weak association between participants level of education and WTP [25]. Achieving secondary level of education was reported to have a positive effect on WTP for HI by Vanithamani [38], as well as Dror et al. [28] But Sana et al. reported a negative effect [35]. They hypothesised that a lower level of education leads to lack of awareness regarding the importance of HI and as such, uneducated people tend to dismiss it. Rahman et al. reported that family size had negative influence in WTP for HI [34]. The opposite finding was reported by Mathiyazhagan and Alam et al. [17, 32]. Higher education emerged as a positive influencing factor on WTP for HI in studies conducted in Iran and Togo [23, 44]. Higher education leads to a better understanding of insurance and finance and may influence participants’ WTP for HI.

SES is usually calculated using various proxies such as wealth index [45, 46], monthly income [47], belonging to a neglected group and so on. Mathiyazhagan reported that participants from backward castes and religious minorities had lowers odds of showing WTP for HI [32]. Similarly, Rahman et al. reported that not owning a privileged card increased the odds of WTP [34]. Considering monthly income, Vanithamani also reported similar findings [38]. One explanation can be that an individual belonging to a family with higher SES is more likely to be financially able and can look for other avenues, like HI, to reduce the health‐related risk. The families with lower SES may not always have that luxury. Comparable findings are reported in other studies [23, 43].

Total five studies reported an association of WTP with family income, seven with occupation and six with MPI. Such associations are well‐established and explained by previous studies conducted in Iran, China, Togo and other settings [23, 41, 43, 44]. Households or individuals with relatively high incomes are likely to be able to contribute or pay for HI and often actively look for other avenues to reduce the health risk [23, 43, 48].

Regarding healthcare‐related determinants, Sarwar and Qureshi reported a weak association between hospitalisation and WTP [36]. Kumar et al. [31] and Dror et al. [28] also reported similar findings. Finally, seven studies reported an association between healthcare utilisation and WTP. It has been suggested that HI has a cyclical relationship with healthcare utilisation. Having HI encourages the use of healthcare services and vice versa [49]. Moreover, having an increased cost of healthcare utilisation also helps individuals to understand the necessity of a financial safety net like HI [30].

This review has several limitations. First, the review was not prospectively registered, and no formal protocol was developed, which may have reduced transparency despite the use of predefined eligibility criteria and a structured review process. Second, the literature search was restricted to PubMed, Google Scholar, BanglaJOL and citation tracking; therefore, studies indexed exclusively in databases, such as Web of Science, EMBASE or CINAHL, may have been missed. Third, only English‐language publications were included, which may have introduced language bias. Fourth, studies were identified from only five of the eight South Asian countries, with most evidence originating from Bangladesh and India, limiting the generalisability of the findings to the entire South Asian region. Nepal was represented by only one eligible WTP study, whereas additional Nepalese literature identified during revision primarily concerns HI enrolment, adherence and policy context rather than empirical WTP estimates [50, 51]. Subgroup analysis by insurance type showed statistically significant differences in pooled WTP estimates between insurance categories, although considerable residual heterogeneity remained within the multi‐study categories. The insurance‐type subgroup analysis was also limited by the small number of studies in some categories, particularly maternal HI and micro HI, which were each represented by a single study, whereas the SHI estimate was based on only two studies. The use of the highest reported WTP proportion when studies reported multiple HI schemes may have introduced an upward bias in the pooled estimate, as this approach preferentially selected the largest reported proportion. The factors associated with WTP were synthesised using a vote‐counting approach. This method summarises the frequency of reported associations but does not account for the magnitude of effect estimates, study quality, or, in some cases, the direction of associations. Consequently, these findings should be interpreted as a qualitative summary of the available evidence rather than a quantitative assessment of the strength or consistency of the associations. Considerable statistical heterogeneity was observed across the meta‐analyses, likely reflecting differences in study populations, HI schemes and WTP elicitation methods. Therefore, the pooled estimates should be interpreted with appropriate caution. A further limitation is that the literature search was completed in February 2024, and therefore studies published after this date were not captured. Given the time elapsed between the search and publication of this review, newer evidence may have emerged that could modify the pooled estimates and conclusions.

## 5 Conclusions

This systematic review of the literature on WTP for HI in South Asian countries has shown that an overwhelming majority of the participants expressed WTP for HI. However, several factors must be considered to tailor HI packages that can attract people and increase participation. The findings of the study may help national policymakers and stakeholders and act as a general outline for introducing and promoting HI in these countries. However, a limited number of studies from several South Asian countries and the observed heterogeneity indicate the need for more robust studies.

## Author Contributions

**Md Fuad Al Fidah** and **Md Abdullah Saeed Khan** conceived and designed the study. **Md Ridwan Islam**, **Sayeda Sumaiya Efa** and Md Fuad Al Fidah were involved in data handling, cleaning and analysis. Md Fuad Al Fidah and Sayeda Sumaiya Efa interpreted the results and wrote the manuscript. Md Ridwan Islam and Md Abdullah Saeed Khan critically reviewed and edited the manuscript. All the authors read and approved the final manuscript for submission.

## Funding

The authors have nothing to report.

## Ethics Statement

The authors have nothing to report.

## Conflicts of Interest

One of the included studies and one of the cited theses were authored by members of the review team. The authors have no other conflicts of interest to declare.

## Supporting information




**Supporting Information File 1**: puh270394‐sup‐0001‐SuppMat.docx

**Supporting Information File 2**: puh270394‐sup‐0002‐SuppMat.pdf

**Supporting Information File 3**: puh270394‐sup‐0003‐SuppMat.doc

## Data Availability

Data, code and other materials used in this systematic review will be shared by the corresponding author on reasonable request.
