# Supplementary material for: Willingness‐to‐Pay for Health Insurance in South Asia: A Systematic Review and Meta‐Analysis
Source: Public Health Chall. 2026 Sep 29;5(4):e70394. doi: 10.1002/puh2.70394 (PMC13620861; doi:10.1002/puh2.70394)
Supplement: Supplementary file 1 [file PUH2-5-e70394-s002.pdf]

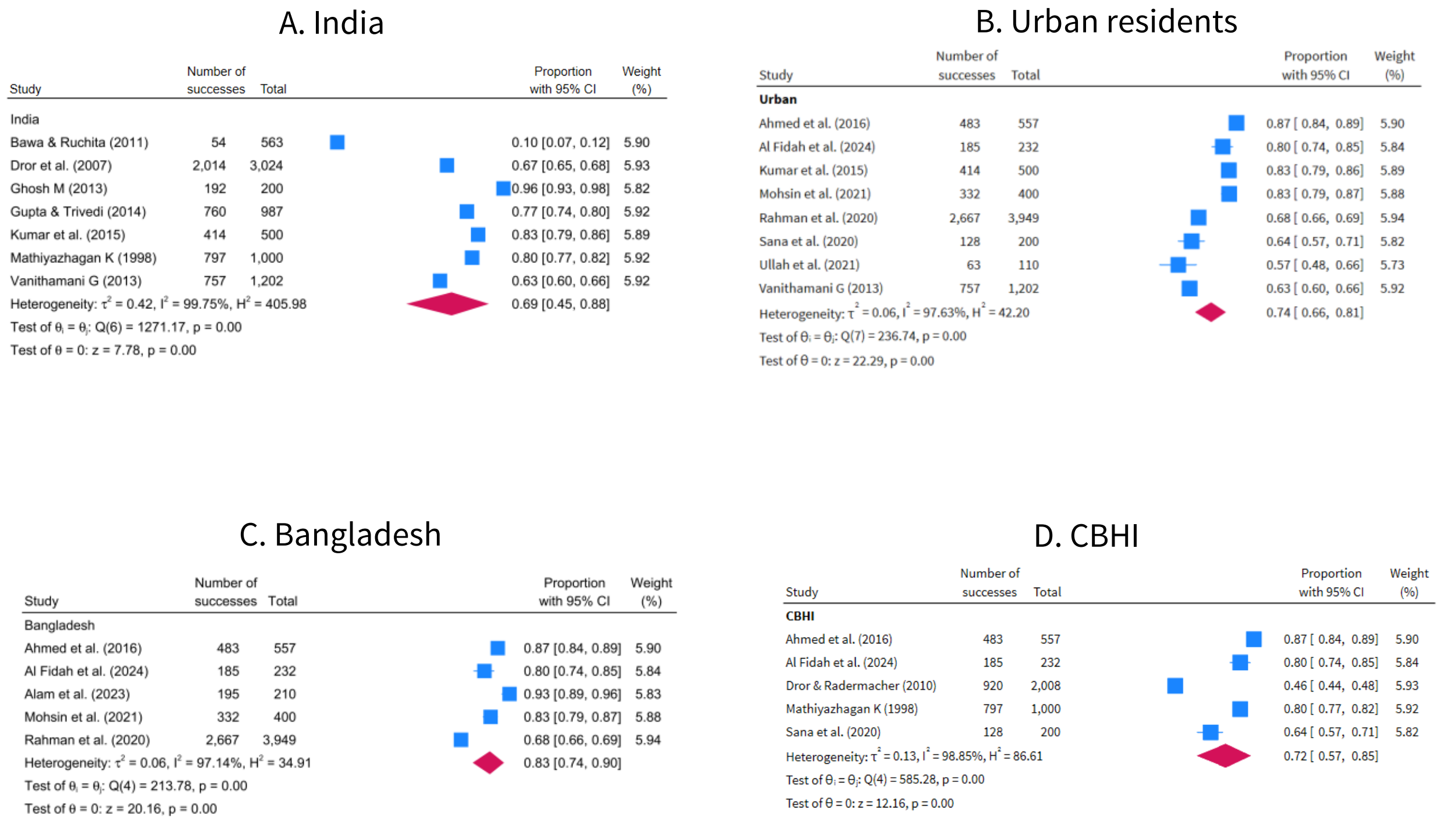

WTP: Willingness-to-pay; HI: Health insurance; CBHI: Community-based health insurance; CI: Confidence interval

Supplementary Figure S1. Subgroup meta-analyses of the proportion of willingness-to-pay for health insurance. Forest plots showing pooled proportion estimates by (A) India, (B) urban residents, (C) Bangladesh, and (D) community-based health insurance (CBHI). All analyses were performed using random-effects REML models

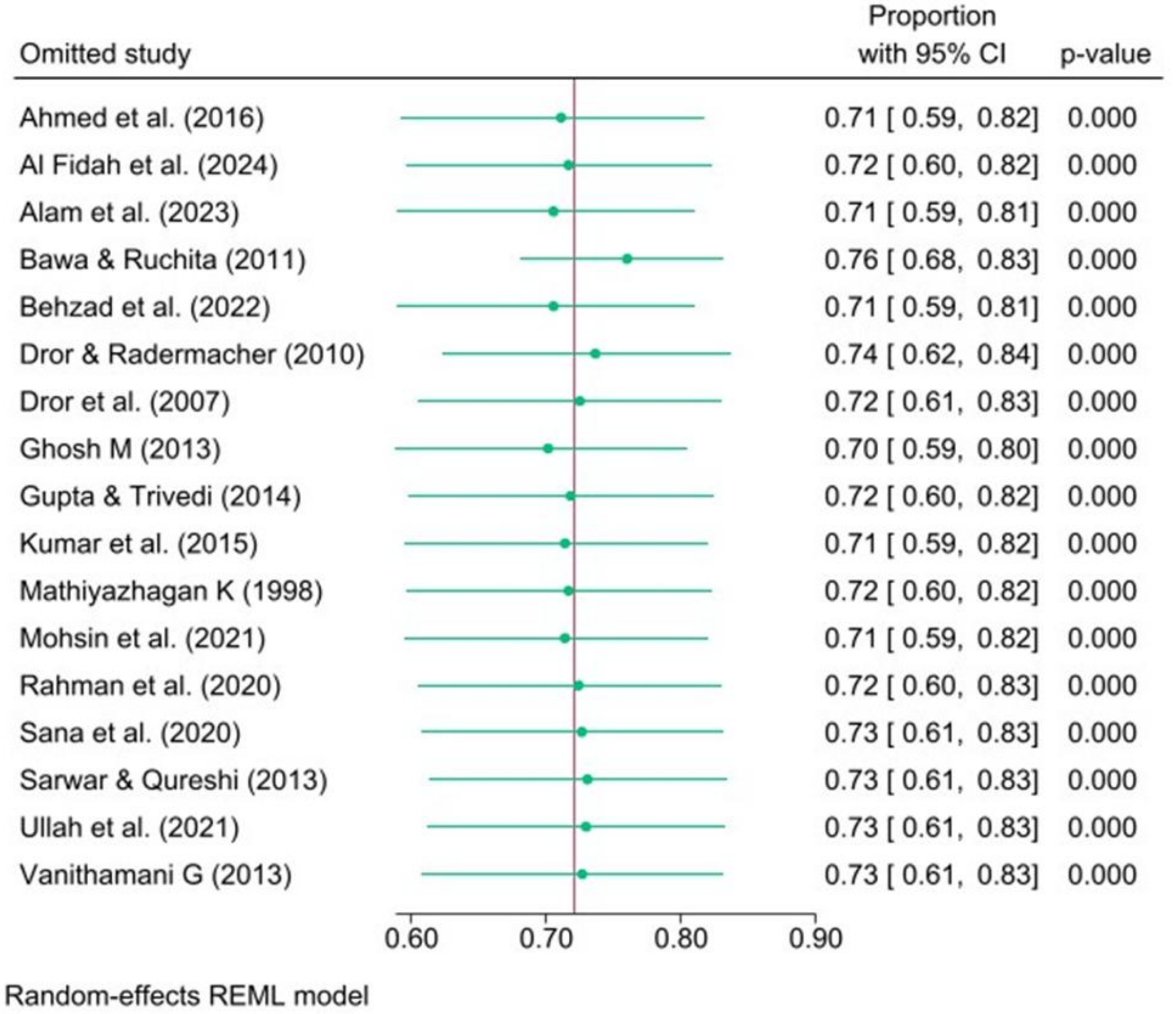

Random-effects REML model; vertical line indicates the overall pooled proportion.

Supplementary Figure S2. Leave-one-out sensitivity analysis of the pooled proportion of willingness-to-pay for health insurance.

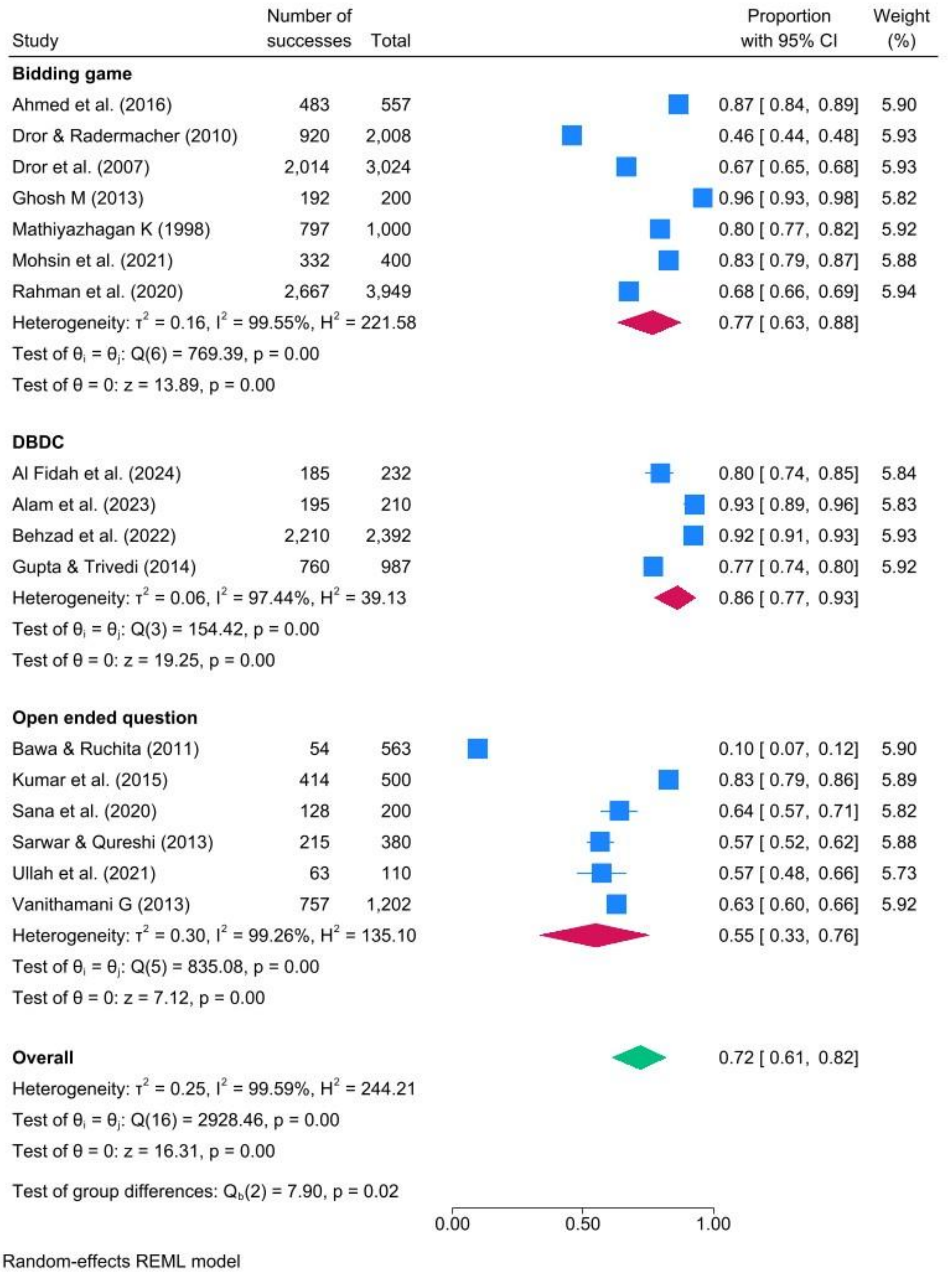

Supplementary Figure S3: Subgroup analysis by WTP elicitation method

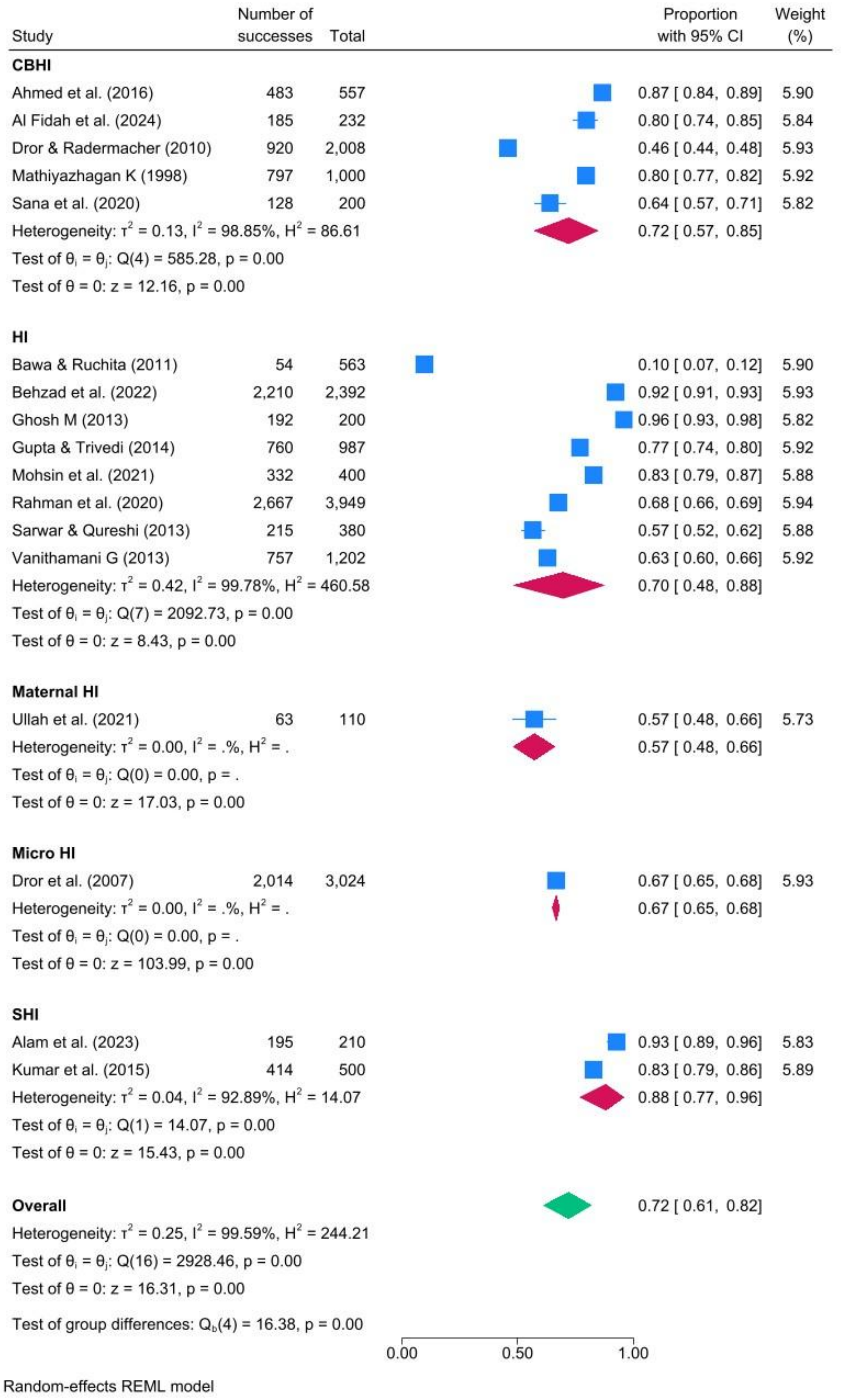

Supplementary Figure S4: Subgroup analysis by insurance type
