# Supplementary material for: Willingness‐to‐Pay for Health Insurance in South Asia: A Systematic Review and Meta‐Analysis
Source: Public Health Chall. 2026 Sep 29;5(4):e70394. doi: 10.1002/puh2.70394 (PMC13620861; doi:10.1002/puh2.70394)
Supplement: Supplementary file 3 [file PUH2-5-e70394-s003.doc]

**Supplementary Table 1:** Search syntax used to identify relevant literature

| **Database** | **Search syntax** |
| --- | --- |
| **PubMed** | ((“Insurance, Health”[Mesh]) AND ((((((((((“Asia, Southern”[Mesh]) OR (Bangladesh)) OR (India)) OR (Pakistan)) OR (Nepal)) OR (Afghanistan)) OR (Bhutan)) ) OR (Maldives)) OR (Sri Lanka))) AND (((Willingness-to-pay) OR (WTP)) OR (“willingness to pay”)) |
| **BanglaJOL** | health insurance |
| **Google scholar** | allintitle:“health insurance” AND “Willingness to pay” AND “Bangladesh” |
|  | allintitle:“health insurance” AND “Willingness to pay” AND “Afghanistan” |
|  | allintitle:“health insurance” AND “Willingness to pay” AND “Bhutan” |
|  | allintitle:“health insurance” AND “Willingness to pay” AND “India” |
|  | allintitle:“health insurance” AND “Willingness to pay” AND “Maldives” |
|  | allintitle:“health insurance” AND “Willingness to pay” AND “Nepal” |
|  | allintitle:“health insurance” AND “Willingness to pay” AND “Pakistan” |
|  | allintitle:“health insurance” AND “Willingness to pay” AND “Sri Lanka” |

**Supplementary table 2: Critical appraisal of the studies**

| **Sl** | **Author (year)** | **Newcastle-Ottawa scale for cross-sectional studies** | | | | **Included/Excluded** |
| --- | --- | --- | --- | --- | --- | --- |
|  |  | **Selection** | **Comparability** | **Outcome** | **Total** |  |
| 1. | Ahmed et al.; 2016 | 4 | 1 | 2 | 7 | Included |
| 2. | Al Fidah et al.; 2024 | 3 | 1 | 2 | 6 | Included |
| 3. | Alam et al.; 2023 | 3 | 1 | 2 | 6 | Included |
| 4. | Bawa and Ruchita; 2011 | 4 | 0 | 2 | 6 | Included |
| 5. | Behzad et al.; 2022 | 3 | 0 | 2 | 5 | Included |
| 6. | Dror and Radermacher; 2010 | 4 | 0 | 2 | 6 | Included |
| 7. | Dror et al.; 2007 | 4 | 1 | 2 | 7 | Included |
| 8. | Ghosh M; 2013 | 2 | 1 | 2 | 5 | Included |
| 9. | Gupta and Trivedi; 2014 | 4 | 1 | 2 | 7 | Included |
| 10. | Kumar et al.; 2015 | 3 | 0 | 2 | 5 | Included |
| 11. | Mathiyazhagan K; 1998 | 4 | 1 | 2 | 7 | Included |
| 12. | Mohsin et al.; 2021 | 4 | 0 | 2 | 6 | Included |
| 13. | Noman SMS; 2021 | 2 | 0 | 2 | 4 | Excluded (NOS score ≤4) |
| 14. | Rahman et al.; 2020 | 4 | 1 | 2 | 7 | Included |
| 15. | Sana et al.; 2020 | 3 | 1 | 2 | 6 | Included |
| 16. | Sarwar and Qureshi; 2013 | 5 | 0 | 1 | 6 | Included |
| 17. | Ullah et al.; 2021 | 4 | 1 | 2 | 7 | Included |
| 18. | Vanithamani G; 2013 | 4 | 1 | 2 | 7 | Included |
| 19 | van der Haar S; 2013 | 1 | 1 | 2 | 4 | Excluded (NOS score ≤4) |
| 20 | Habib SS; 2021 | 2 | 0 | 1 | 3 | Excluded (NOS score ≤4) |
